# Lymphome T/NK extra-nasal à localisation colique primitif: à propos d’un cas

**DOI:** 10.11604/pamj.2017.26.112.10304

**Published:** 2017-03-01

**Authors:** Salma Fares, Mouna Lamchahab, Myriem Aniba, Ghizlane Lembarki, Nadia Mousalli, Meriem Regragui, Mehdi Karkouri, Asmaa Quessar

**Affiliations:** 1 Service d’Hématologie et d’Oncologie Pédiatrique, Hôpital 20 Août 1953, CHU Ibn Rochd, Casablanca, Maroc; 2 Service de Radiologie de l’Hôpital 20 Août 1953, CHU Ibn Rochd, Casablanca, Maroc; 3 Service Central d’Anatomie Pathologique, CHU Ibn Rochd, Casablanca, Maroc

**Keywords:** Lymphome T/NK, extra-nasal, colique, entéropathie, T/NK lymphoma, extra-nasal, colic, enteropathy

## Abstract

Le lymphome T/NK intestinal primitif est une entité extrêmement rare dont le diagnostic précoce est souvent difficile. Pour mieux comprendre cette entité, nous rapportons le cas d’un patient de 43 ans diagnostiqué avec un lymphome T/NK colique primitif localisé et isolé sans entéropathie associée, ayant été traité par 3 cycles (AspaMetDex) avec un échec à l’évaluation et la survenue du décès au cours du traitement dans le tableau d’un abdomen aigu. Le lymphome T/NK intestinal primitif atteint le plus souvent le sujet jeune avec un pronostic péjoratif. En raison des caractères cliniques et endoscopiques non spécifiques, il est difficile de distinguer entre un lymphome T/NK intestinal et les troubles intestinaux inflammatoires ou infectieuses. Les données histopathologiques et immunohistochimiques ainsi que l’étude de l’ADN permettent de redresser le diagnostic et de classer ce lymphome selon l’EuropeanEnteropathy-Type Intestinal T-CellLymphoma (ETL).

## Abstract (en)

To better understand this cancer, we here report the case of a 43-year old patient diagnosed with localized and isolated primary colonic NK/T-cell lymphoma without associated enteropathy, treated wih 3 cycles of AspaMetDex with a poor response who died during treatment with a clinical picture of acute abdomen. Primary intestinal NK/T-cell lymphoma most commonly affects the young subject with poor prognosis. It is difficult to distinguish between intestinal NK/T-cell lymphoma and inflammatory or infectious intestinal disorders because of its non-specific clinical and endoscopic features. The histopathological and immunohistochemical data as well as the study of DNA allow to adjust the diagnosis and to classify this lymphoma according the European Enteropathy type T-cell lymphoma (ETL).

## Introduction

Selon l’OMS, la classification des lymphomes englobe un large éventail d’entités pathologiques. Le lymphome à cellules T/NK représente environ 3% de la totalité des lymphomes non hodgkiniens (LNH) [1]. En Asie, le lymphome à cellules T/NK représente entre 2 et 8% de l’ensemble des LNH [2–4] alors qu’en Europe sa prévalence ne dépasse pas 2% [2]. Le lymphome à cellules T/NK extra-nodal est un sous-groupe de lymphomes T cytotoxiques ou à cellules NK ayant un spectre morphologique multiple qui provient de l’extérieur du ganglion lymphatique. Les cellules NK sont des cellules non B/non T avec un rôle bien reconnu dans l’immunité innée. Leur nature cytotoxique leur permet d’éradiquer les différents types de virus ainsi que les cellules tumorales. Récemment, leur rôle dans l’immunité adaptative a été bien élucidé à travers leur fonction d’effecteur de production des cytokines [5]. Le lymphome à cellules T/NK est un lymphome agressif caractérisé par une prolifération lymphoïde atypique angiocentrique ou angiodestructive associée à une nécrose [6]. Ces lésions sont presque toujours associées à l’EBV et montrent une positivité au marqueur CD56. Selon leur site, ils sont classés en lymphome à cellules T/NK nodal (NNTCL) et extranodal (ENTCL) [1]. Le lymphome à cellules T/NK intestinal primitif est défini comme un lymphome extra-nodal à développement intestinal avec la majorité de la masse tumorale localisée en intestinal ; cette entité est extrêmement rare. Depuis le diagnostic précoce de ce lymphome, il présente toujours des difficultés pour les cliniciens, le traitement approprié pourrait être retardé. Le diagnostic différentiel de tels cas comprennent habituellement : le lymphome à cellules T associé à une entéropathie (EATL) [7], le lymphome à cellules T gamma-delta [8] et le lymphome anaplasique à grandes cellules (ALCL) [9]. Le traitement de ce lymphome agressif consiste en une polychimiothérapie systémique et éventuellement un traitement myéloablatif. Objectif: pour mieux comprendre cette entité, nous rapportons le cas d’un lymphome à cellules T/NK intestinal primitif avec des caractéristiques pathologiques d’une entéropathie sans association à une maladie cœliaque.

## Patient et observation

**Figure 1:**
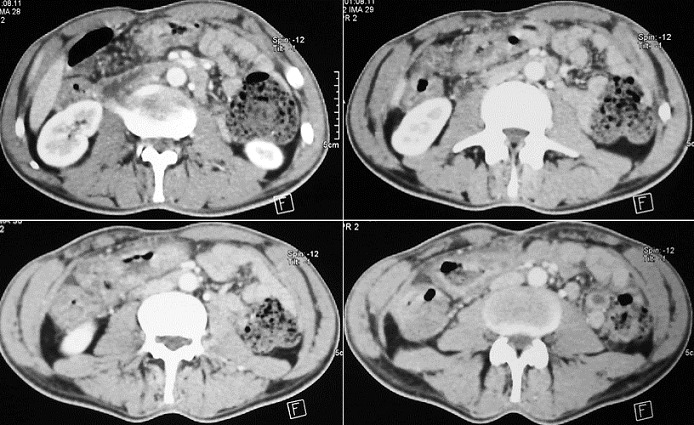
Épaississement pariétal circonférenciel irrégulier du colon transverse, étendu sur 6 cm de hauteur, avec infiltration nodulaire et en flammèche de la graisse mésocolique. Il s’y associe la présence d’adénopathies péricoliques transverses

**Figure 2:**
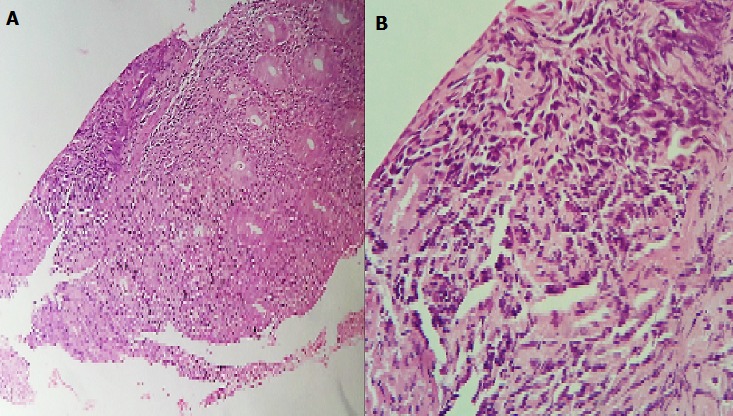
Biopsie colique colorée à l’hématoxyline- éosine: A (*100) Revêtement partiellement ulcéré, infiltrat lymphoïde diffus. B (*400) Infiltrat lymphoïde fait de cellules atypiques de taille moyenne à grande à noyaux irréguliers hyperchromatiques

**Figure 3:**
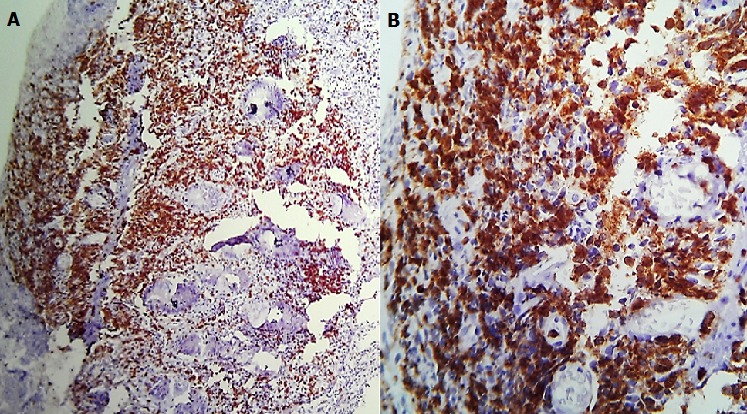
Immunohistochimie par l’anticorps antiCD3: C (*100) D (*400): Marquage cytoplasmique diffus et intense

**Figure 4:**
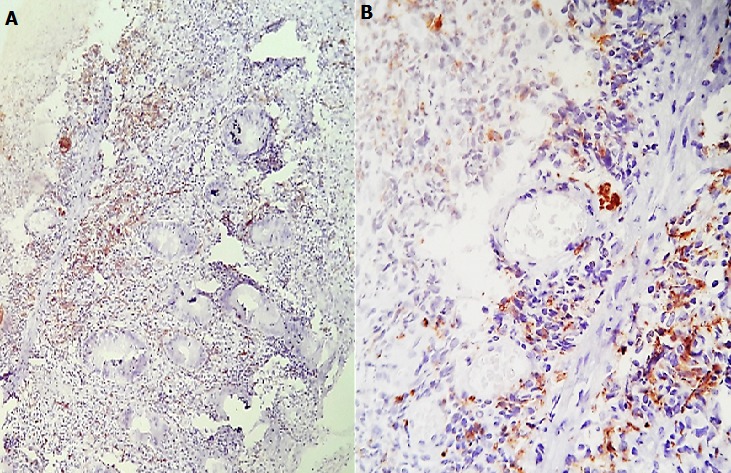
Immunohistochimie par le CD56: E (*100) F (*400): Marquage membranaire des cellules atypiques

**Figure 5:**
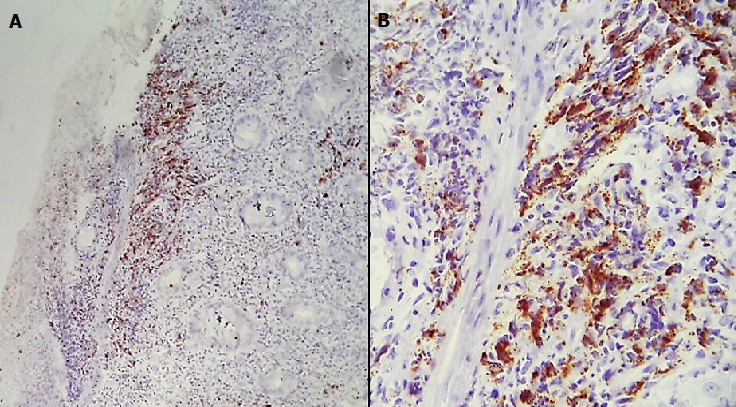
Immunohistochimie par l’anticorps anti Granzyme B: Marquage cytoplasmique G (*100) H (*400)

Un homme âgé de 43 ans, sans notion de maladie de malabsorption ou de maladie cœliaque diagnostiquées auparavant, admis au service d’Hématologie pour une symptomatologie qui semble remonter à 7 mois par l’installation de douleurs abdominales, de diarrhées glairosanglantes, associées à une fièvre, sueurs et à un amaigrissement chiffré à 18 kg en 2 mois. L’examen physique retrouvait un patient avec un ECOG PS à 2, un abdomen sensible sans masse palpable et un toucher rectal qui retrouvait des stigmates de rectorragies. La TDM abdominale a montré un processus du colon transverse mesurant 6 cm de hauteur avec une carcinose péritonéale localisée et présence d’adénopathies locorégionales (Figure 1). La colonoscopie avait montré une masse bourgeonnante irrégulière, hautement suspecte de malignité au niveau du colon transverse. La biopsie colique avait montré un LNH T/NK colique CD3+ cytoplasmique, CD4+, CD8+, CD56+, Granzyme B+, EBV+ (Figure 2, Figure 3, Figure 4, Figure 5). Le bilan d’extension incluant une TDM cervico-thoraco-abdomino-pelvienne n’avait pas montré de localisations en dehors de la masse colique; la biopsie ostéo-médullaire n’a pas montré d’infiltration lymphomateuse; le taux de LDH était normal; les sérologies de l’EBV ont montré une immunisation antérieure avec une positivité des IgG et une négativité des IgM. L’examen ORL ainsi que les scanners cérébral et facial étaient normaux. La recherche d’une entéropathie notamment la recherche des anticorps anti-gliadines et anti-endomysium type IgA et IgG s’est révélée négative, et la biopsie colique a montré un aspect de rectite inflammatoire sans arguments suffisants pour évoquer une entéropathie. Le bilan pré-thérapeutique notamment l’échocardiographie n’a pas montré d’anomalies. Le patient a été classé Stade IIE selon la classification Ann Arbor avec un International Pronostic Index (IPI) à 0. Sur le plan thérapeutique, le patient a reçu 3 cycles (AspaMetDex) (J1-J21) avec Méthotrexate à 3g/m2 (J1)-Dexaméthasone 40 mg/j (J1-J4)- L-asparaginase 6000 UI/m2 (J2, J4, J6, J8). Durant le traitement, le patient a développé un diabète cortico-induit pour lequel il a été mis sous insulinothérapie et une toxicité hématologique et muqueuse grade IV secondaire au Méthotrexate. Sur le plan clinique, le patient gardait toujours les douleurs abdominales avec une légère régression des diarrhées glairo-sanglantes. Le scanner abdominal réalisé à 2 cures de chimiothérapie a montré un aspect stable de la lésion colique et la biopsie colique a montré le LNH T/NK colique déjà diagnostiqué. L’évolution a été marquée par la survenue du décès dans le tableau d’un abdomen aigu probablement secondaire à une perforation au 10^ème^ jour de la 3^ème^ cure de chimiothérapie.

## Discussion

Les lymphomes intestinaux à cellules T et à cellules NK représente 5.2 à 14.7% de l’ensemble des lymphomes primitifs du tractus gastro-intestinal [10, 11]. Cette entité inclus le lymphome à cellules T associé à une entéropathie (EATL), le lymphome anaplasique à grandes cellules (ALCL), le lymphome à cellules T/NK extra-nodal (ENTCL) et le lymphome à cellules T périphérique (NOS) [12]. Le lymphome à cellules T/NK extra-nodal (ENTCL) est prédominant en Asie et en Amérique centrale et du Sud et atteint surtout les sujets jeunes avec un pic de morbidité à l’âge de 31-40 ans comme ce qui est le cas de notre patient avec un pronostic souvent pauvre [4], alors que sa prévalence reste relativement faible en Europe et en Amérique du Nord et survient chez les sujets plus âgés [9]. La présentation clinique chez notre patient était compatible avec ceux rapportés dans la littérature [13, 14]. Les symptômes révélateurs peuvent être subdivisés en signes secondaires au processus tumoral (douleur abdominale, diarrhée, occlusion intestinale), à la destruction tissulaire (perforation intestinale, péritonite et rectorragies) et en signes généraux (fièvre et amaigrissement).La colonoscopie chez notre patient a montré une masse bourgeonnante irrégulière au niveau du colon transverse, dans une étude chinoise sur 25 cas, 16 patients avaient une localisation colique et 4 patients avaient des masses tumorales [15]. En raison de la disparité des observations cliniques et endoscopiques des lymphomes intestinaux primitifs à cellules T/NK, la distinction entre les troubles inflammatoires, infectieux, granulomateux et lymphomateux restent difficile. Le diagnostic histologique de cette entité peut être confondu avec un désordre inflammatoire ou infectieux. Par conséquent, une forte suspicion clinique de malignité avec une histologie négative ne doit écarter le diagnostic et d’autres biopsies répétées et profondes sont fortement recommandées au cours du traitement et du suivi, et dans le cas échéant une laparotomie exploratrice qui peut être indiquée dans les diagnostics précoces. L’immunohistochimie a retrouvé l’expression des antigènes CD3+ cytoplasmique, CD56+, Granzyme B+, EBV+, ce phénotype concorde avec les données de la littérature [16]. Une étude a reporté que les patients ayant le statut EBV négatif avaient une survie globale un peu plus longue que ceux EBV positif, ce qui indique que l’infection à l’EBV peut être considéré comme un facteur pronostique négatif indépendant dans les lymphomes à cellules T/NK [17], deux autres études ont démontré dans le même sens que l’infection à l’EBV peut être supposée comme un facteur de risque de déclenchement de la genèse tumorale [18, 19]. Les données du suivi de la série de Zheng et al, ont indiqué que le lymphome primitif à cellules T/NK avait un pronostic péjoratif avec une survie médiane de 7 mois, 64% des patients ont été diagnostiqués à un stade avancé et 60% des patients avaient une altération des capacités de vie par le lymphome avant le traitement [15]. Chez notre patient, le lymphome était localisé avec un IPI à 0 mais le patient est décédé après 5 mois de la date du diagnostic, ce qui suggère l’existence d’autres facteurs de risque indépendants de l’IPI dans ce type de lymphome. Une étude japonaise sur 30 cas a suggéré que la morphologie cellulaire ainsi que l’invasion angiocentrique peuvent avoir une conséquence sur le pronostic du lymphome intestinal primitif à cellules T/NK [19]. Dans notre cas, le patient a reçu des cycles (AspaMetDex) en première ligne avec un échec à 3 cycles du fait de la toxicité importante du protocole SMILE (Dexaméthasone, Méthotrexate, Ifosfamide, L-asparaginase, Etoposide) et de la précarité de son état général. Un essai clinique phase II chez 19 patients avec un lymphome à cellules T/NK type nasal en rechute ou réfractaire ont reçu le protocole (AspaMetDex) avec une rémission complète à 61% et une rechute chez 4 patients, la survie globale médiane était estimée à 1 an avec une durée médiane de réponse à 12 mois [20].

## Conclusion

Le lymphome T/NK intestinal primitif atteint le plus souvent les sujets jeunes avec pronostic péjoratif. En raison des caractères cliniques et endoscopiques non spécifiques, il est difficile de distinguer entre un lymphome T/NK intestinal et les troubles intestinaux inflammatoires ou infectieuses. L’histopathologie, l’immunohistochimie et l’étude de l’ADN jouent un rôle clé dans le diagnostic différentiel et permet de classer le lymphome selon l’EuropeanEnteropathy-Type Intestinal T-CellLymphoma (ETL).
